# Journey Through Healthcare of People With Complications of Type 2 Diabetes: A Qualitative Study of Lived Experiences

**DOI:** 10.5334/ijic.7604

**Published:** 2024-05-21

**Authors:** Stijn De Baets, Katrien Danhieux, Eveline Dirinck, Bruno Lapauw, Edwin Wouters, Roy Remmen, Josefien van Olmen

**Affiliations:** 1 Ghent University, Faculty of Medicine and Health sciences, department of rehabilitation sciences, Occupational therapy research group, Ghent, Belgium; 2 Vrije Universiteit Brussel, Faculty of Medicine and Pharmacy, Frailty in ageing research group, Brussels, Belgium; 3 Antwerp University, Faculty of Medicine and Health Sciences, Department of Family Medicine and Population Health, Antwerp, Belgium; 4 Antwerp University Hospital, Department of Endocrinology, Diabetology and metabolic disease, Antwerp, Belgium; 5 Ghent University Hospital, Department of Endocrinology, Ghent, Belgium; 6 Antwerp University, Faculty of Social Sciences, Department of Sociology, Antwerp, Belgium

**Keywords:** access to care, Type 2 diabetes, high income countries, financial barriers, self-management

## Abstract

**Background:**

Despite its overall good performance, the Belgium healthcare system scores less well in providing equal access to healthcare compared to other European countries. This increases the risk of people worse off to receive late diagnosis and to get complications of chronic diseases.

**Methods:**

This study aims to achieve a deeper understanding of how people with complications of a chronic disease – diabetes type 2 – experience care in the Belgium health system through semi-structured interviews with extreme case study sampling of people with advanced diabetes, and inductive analysis.

**Results:**

The results show that most respondents were diagnosed late in the course of their disease. There are variations in treatment and type of provider. People appreciate the personal and long-lasting contact with a medical doctor, while the contact with and role of paramedical providers was less recognized. Disease management has a significant impact on their financial budget and some respondents experienced barriers to obtain additional financial support.

**Discussion:**

Non-medical costs are not reimbursed, presenting a high burden to people. Self-management is tedious and hampered by other worries that people may have, such as financial constraints and coping with important life events. To conclude this study highlighted the need to improve diabetes screening. We suggest to enhance the role of paramedical professionals, integrate a social care worker, reduce financial constraints, and increase health literacy through more patient-centered, goal-oriented care.

## Introduction

Type 2 diabetes mellitus (T2DM) related complications like cardiovascular and neurological complications and other comorbidities have a great socio-economic impact and reduce quality of life. Health systems can positively influence the awareness, treatment, adherence and control of the disease. Healthcare that is organized in an integrated way using multidisciplinary teams of healthcare professionals and focusing on patient empowerment increase access and quality of care and the support to self-management [1,2].

To reduce the economic burden of T2DM and to increase quality of care, Belgium, like many high-income countries, implemented care pathways. Three care pathways for T2DM exist: one for people getting at least three injections of insulin per day (the diabetes convention), one for people taking or considering injections of insulin or an injectable incretin mimetic (the diabetes care trajectory) and one for patients on oral treatment or without antidiabetic treatment (the diabetes pre-trajectory). These programs include (depending on the program) annual consultations with a general practitioner (GP), a nurse diabetes educator and an endocrinologist/diabetologist and self-management material which is largely reimbursed. Effect evaluation studies suggest that each pathway leads to a higher number of people being properly managed [3,4]. To ensure access to the health system, including the care trajectories, there are extra financial protection mechanisms for people in vulnerable situations to reduce their costs. This status of ‘increased reimbursement’ implies entitlement to higher reimbursement of costs. This status is granted to people with an income below a certain level. Some other financial mechanisms are a maximum billing system and the third-payer measure, which are both granted to patients with a number of chronic illnesses [5].

Despite the overall good performance of the health care system, the system does not work equally well for the entire population of Belgium [6]. Belgium is in the top ten spenders on health in Europe and out of pocket payments are about 18,2 percent, which is more than its neighboring countries and the EU mean [6]. 2.4% of the adult population reported unmet needs for medical care due to financial reasons. In the lowest income quintile, this raises up to 6.7% [7]. Similarly, 4.3% of people report unmet needs among the 20% people with the lowest levels of education versus 1.3% of people in the highest education quintile [6]. So, with regards to equitable access to care for vulnerable groups, Belgium underperforms in relation to comparable OECD countries [7,8]. There is also room for improvement in the organization of care, such as tailoring care to the specific population needs, strengthening role of primary care, and financial reform to facilitate collaboration [9]. These gaps in access and quality can lead to serious complications [1].

**Figure 1:**
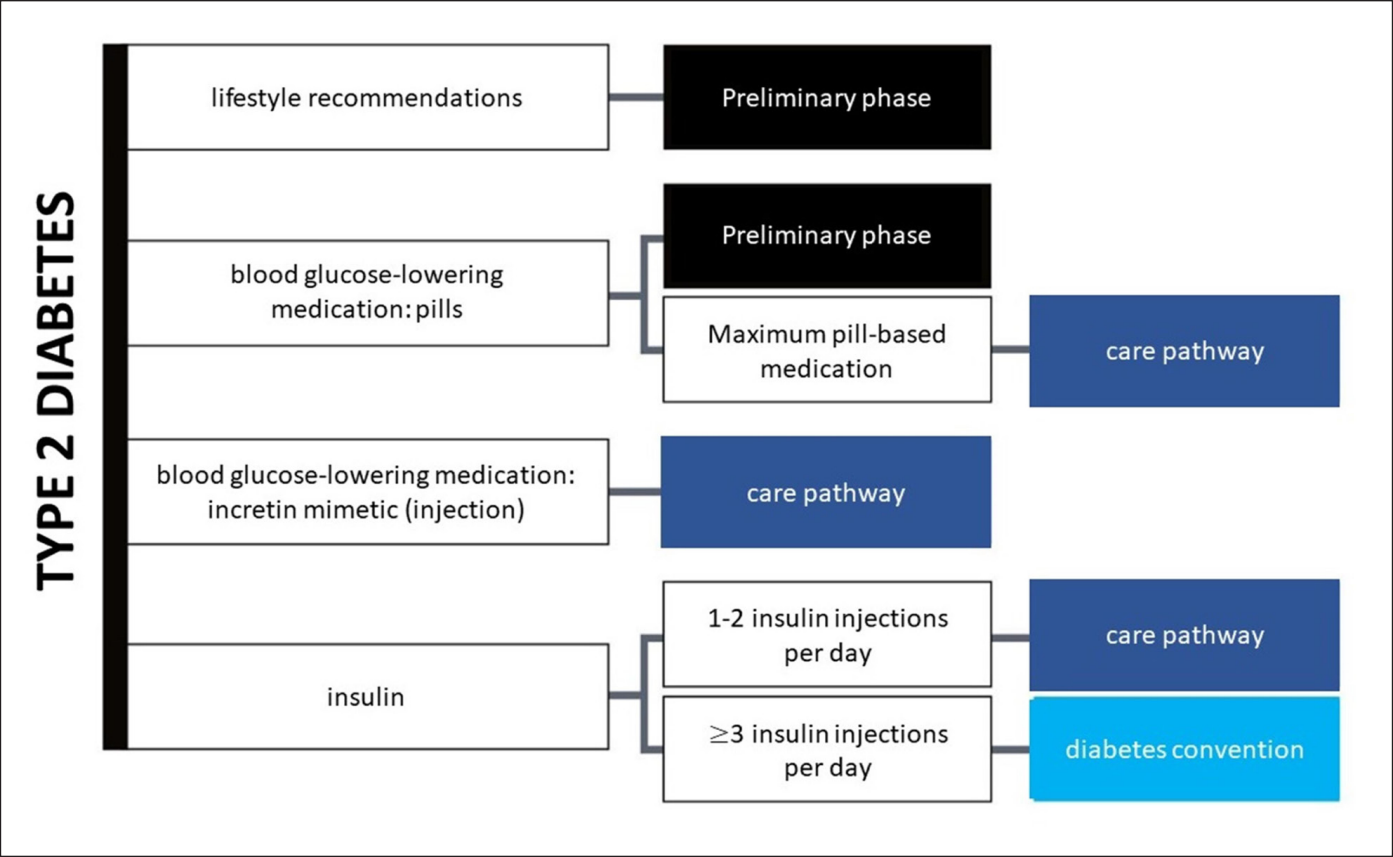
T2DM care systems and facilities in Belgium (www.diabetes.be).

The studies above take the macro level data of the health system as a focus. Patient journeys are a relatively new concept that identify the pathways of services patients go through having a chronic disease like T2DM. To date, to our knowledge, there are no studies that examine the patient journeys and (unmet) needs from the perspective of the users of health care in Belgium [10]. This would provide insight on why this generally well-performing system fails to engage with all people with care needs in the most effective way. This study therefore aims to achieve a deeper understanding of how patients with a typical chronic disease like T2DM with complications experience the care they receive. How do people with T2DM with complications in Belgium health services experience the available health care and support to them? What barriers and/or encouragements to care and self-management do they perceive? Both the methods we used and our findings can help researchers of other health care systems understand the complex interactions when eliciting the problems encountered by patients with longstanding chronic conditions in their journey through the health systems. In Figure 1 a brief overview is provided of the services provided to T2DM patients.

## Methods

A qualitative research using semi-structured interviews with open-ended questions was conducted to explore the experiences with the health system of people with T2D with complications. This study is part of the research project: SCale-Up diaBetes and hYpertension care (SCUBY), which aims to scale-up integrated care through the development and evaluation of roadmap strategies in different types of health care systems in Belgium, Cambodia and Slovenia [11].

The inclusion criteria were: age ≥ 50 years, having complications as a result of T2DM, and speaking Dutch, French or English. Complications were defined as long-term complications of T2D and included being treated for cardiovascular complications, renal failure, visual impairment or foot problems. The sampling strategy is a form of extreme case sampling, because of its purpose to select respondents that are not representative for the group of people with T2D, but instead represent a particular (extreme) segment of the population [12]. Within this group, maximum variation in the following aspects was strived for: urban and rural; followed up in primary or secondary care; with and without a migration background; and different socio-economic strata and a variety of the complications mentioned above. Recruitment was done in two university hospitals, two peripheral hospitals and two primary care practices, in order to reach variation of care used. The participants were approached by their medical doctor and asked if they wanted to participate. Afterwards, potential participants were contacted by the interviewer, and the informed consent procedure followed. Data collection continued until saturation of the data was reached and no new themes emerged from the interviews [12,13]. Specifically for this project, the dynamic, non-chronological organization involves insights from initial analyses informing subsequent data collection, and vice versa. This iterative, forwards-and-backwards approach aimed to ensure our exploration of themes reaches a point of saturation.

Most interviews took place in the hospital, following the outpatient consultation with the medical doctor, at the primary care physician’s practice or at the home of the participants. After the start of the lockdown in Belgium following the COVID-19 pandemic, some interviews were then conducted by telephone or videoconferencing. Participants were given the choice to be assisted by their family members or not.

The interviews started with the open-ended question ‘Could you tell me something about the impact of T2DM on your life?’ Thereafter, the participants were encouraged to give a detailed account of their subjective experiences with their chronic disease. Probing questions were used to deepen the answers and were adapted to the participants’ responses. Specifically, the researchers focused on the process of the T2DM diagnosis, the turning points in the worsening of the disease and what possibly prevented them from getting appropriate care.

**Figure 2:**
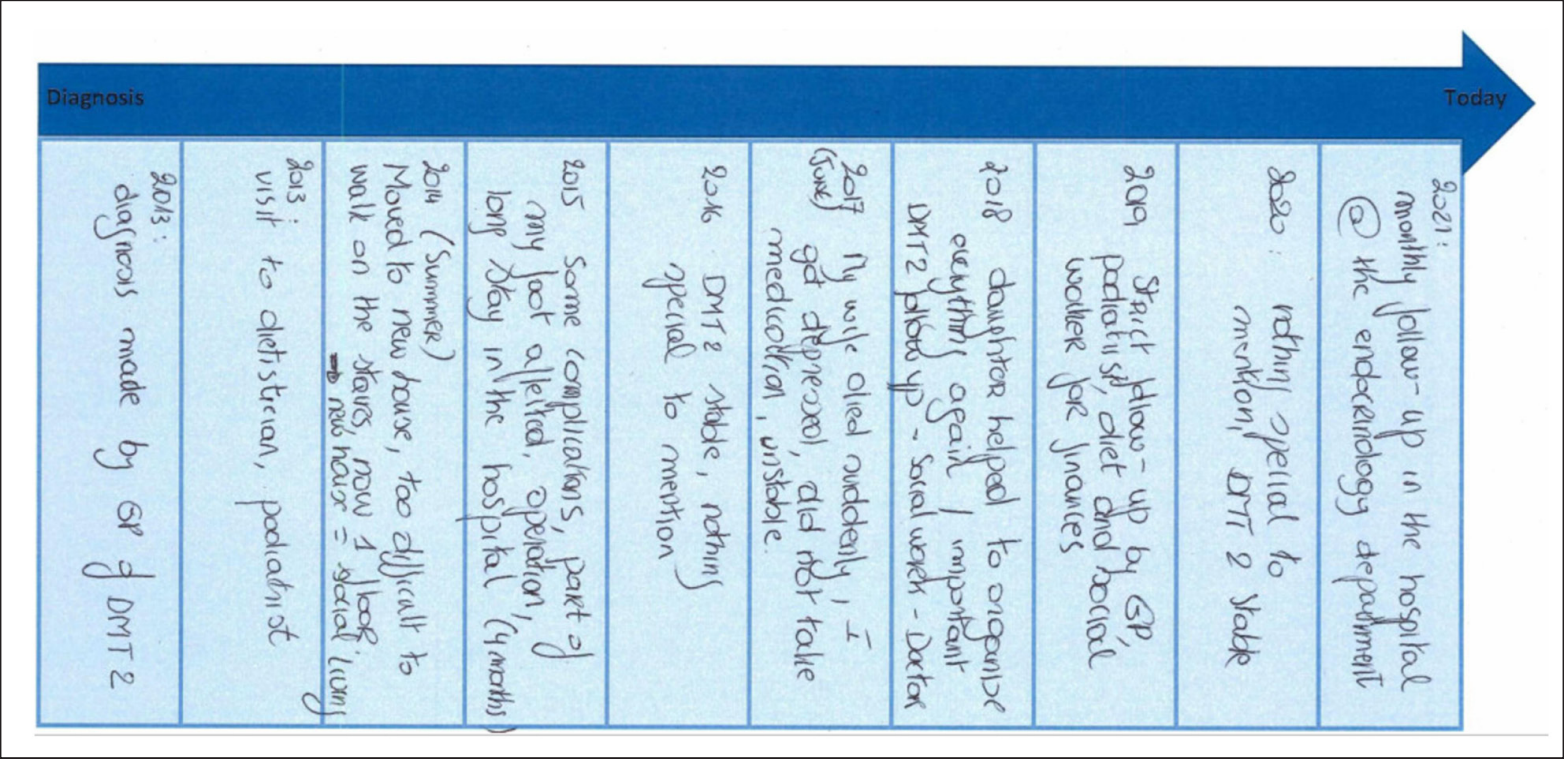
Example of a biographical–visual timeline.

A biographical approach was used to create an overview of the broader context (e.g. the diagnostic process, the care received from health professionals, contextual and personal factors that influence the T2DM care) to elicit the views and experiences of the participants [14,15]. This allowed the creation of a visual timeline drawn together with the participants in order to structure their memories (Figure 2). The interview started from the timeline, with probing questions based upon an interview guide and a topic list (Table 1). Focus in the interviews was on turning points that were remembered as important for the course of their illness. These turning points were indicated in the visual timeline (see example in Figure 2). The interviews were recorded and transcribed verbatim. The patient journeys were collected during the interviews. They showed great diversity. In the analysis we used them in the development of the themes.

**Table 1:** Characteristics of the participants and of interview methods.


VARIABLE	VALUE	N	PER CENT

Gender	Male	18	64%

	Female	10	36%

Social status	Married	15	54%

	Widow	8	29%

	Living together	2	7%

	Divorced	2	7%

	Single	1	4%

Nationality	Belgian	24	86%

	Non-Belgian	4	14%

Residence	Urban	14	50%

	Rural	14	50%

Complications	Renal	16	57%

	Ophthalmological	12	43%

	Foot problems	9	32%

	Cardiac	8	29%

	Amputation lower limb	7	25%

	Stroke	4	14%

	Neuropathy	4	14%

Interview method	Live	22	79%

	Phone	5	18%

	Videoconference	1	4%

Location of live interview (n=22)	Outpatient ward of hospital	13	46%

	General practitioner	6	21%

	Home	3	11%


Inductive thematic analysis was done, led by the first and second authors with regular discussion with the last author [16]. The analysis was conducted in three phases. In the first phase, the first two authors familiarized themselves with the data by reading and re-reading the transcripts of the interviews. Initial ideas were noted and shared among the two first authors and the last author. In the second phase, interesting features of the data were systematically coded by the first author in an iterative process, starting with the first 10 transcribed interviews, discussing it with the co-authors and continuing with the other interviews. This process continued until the entire dataset was coded. No new information was gained after 26 interviews. The last two interviews were used to check whether the same information was discovered. In the third phase, the initial codes were discussed among the first, second and last authors. Additional elements were added by the second author, and the similarities in the codes were collated into potential themes. This process resulted in a thematic map. Finally, in the fourth phase, the specifics of each theme were refined [17]. Note that the above phases did not occur linearly: there was back and forth movement among the phases.

The Ethics Committees of the Universities of Antwerp & Ghent approved the study (Belgian reference number B3002020000251) and informed consent was obtained from all participants.

## Results

Twenty-eight people, aged between 50 and 83 years, were included in this study. The mean age was 70 years and mean time since diagnosis was 20 years. The duration of the interviews varied from 14 to 87 minutes, with an average of 38 minutes. In some cases, a family member (partner or child) joined the interview. Detailed characteristics of the participants and the interviews are shown in Table 1.

**Figure 3:**
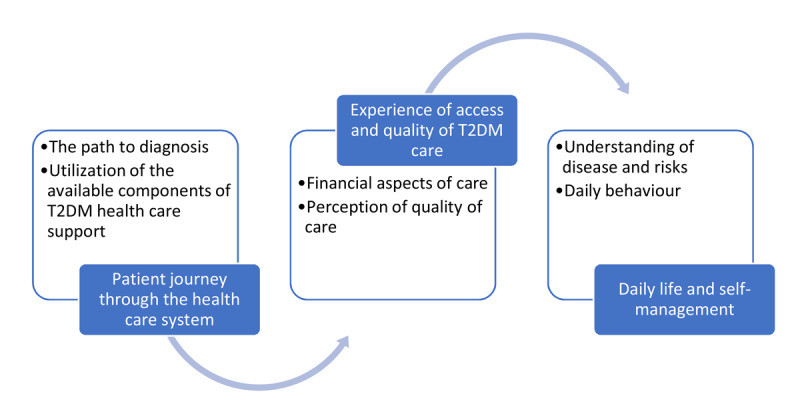
Chronological overview of the themes and subthemes.

**The inductive thematic process.** The first phase led to three themes as a general construct: personal expenses linked to T2DM and the consequences of it on the disease; variation in care pathways; and the impact of T2DM on performing daily activities. Further in-depth analyses revealed the following subtopics: the importance of financial aspects; support in self-management; care process; psychosocial aspects that influence daily life; the importance of lifestyle; quality of the delivered care; and the distance to care facilities. In the third phase, the topics were synthesized into three overarching themes (Figure 3 and annex 2).

### Patient journey through the health care system

The respondents had made very different journeys through the health care system, from being followed up by the GP from diagnosis onwards for many years to being diagnosed at the emergency department and follow up in the academic hospital. Most respondents were diagnosed when their disease was already in advanced stage, namely with complaints or even complications. (*“Well, I didn’t know I had diabetes, that’s how it started. I entered the hospital with a black toe and there they said: “Yes, sir, you do have diabetes.”- P14)*. However, some took part in routine or occasional screening that revealed increased glucose. (‘*I was diagnosed with diabetes when doing a regular medical check-up at work, and then I went to the GP.*’). The involvement of medical specialists next to general practitioners was not common for all respondents; some people had been followed up by a GP for many years before being referred (‘*I have known my GP for 45 years. And he knows me very well too. He was the one who discovered my diabetes in the first place. Every two months I had a blood test and he saw that it was okay, and in some months, we managed to stabilize it. But only with medication. And it lasted all the time like that. But then a while ago he told me that I had to see a diabetologist.’ (P4))*. The intensity of care varied, depending on the medical condition of the respondent. While some patients needed home care by a nurse multiple times a day, others were still rather independent, despite their complications. For some patients, their GP was their central point of contact, whereas others relied on their diabetologist, although they had to visit the GP yearly to follow the regulations of the diabetes convention. (‘*I don’t get free testing material if I don’t visit my GP yearly. But I am three times a month in the University Hospital, with a physician and I don’t really need a GP.’ (P26))*. Most respondents also consulted a specialist, specifically a cardiologist and/or an ophthalmologist. Paramedical services such as dieticians, psychologists and podiatrists were consulted rather occasionally and not by all respondents.

### Experience of access and perceptions of quality of T2DM care

The interviewees overall agreed on the large impact of the care on their financial situation. Estimations of healthcare expenditures varied between 20% and 70% of their (self-reported) income on care-related expenses *(‘It’s not 50% of my income, but for sure 25%, since I don’t buy orthopedic shoes every year, but I often have for example to buy new elastic bandages for my legs and medication’ (p19))*.

Some participants indicated to receive additional disability benefits for disability or informal care, or benefits in kind, for instance on adaptations in the home situation. *(‘I have a pension of 1058 euros and I have 130 euros for informal care and that is how I pay for my nursing and other expenses. I have just under 200 euros from the disability benefit, which is also added to my income, but I know people who have even less.’* (p11)). People who are included in the national diabetes convention get most of their direct medical expenses reimbursed. However, participants indicated that they made additional non-medical expenses that were not (fully) reimbursed, such as orthopedic shoes, foot wound care, transport, adapted diet, dental care, podiatrist, specific medication, hospital admissions, etc. (‘*The biggest cost for me is the taxi, there and back. If I have to go to the hospital by taxi, it always costs me 25 to 30 euros. If you say once a month ok, but if you have to go to the doctor often. All those little things add up*.’ (p11)). Other interviewees did not know some specific care was reimbursed for patients with T2DM. *(‘I didn’t know that you could have the podiatrist come every year and that you could get a reduction of EUR 5 for that.’ (p11))*.

Participants struggle with the administration burden of getting reimbursement and benefits. They need to submit requests for the different type of special exemptions and benefits they are entitled to. (‘*Lots of papers, yes, for the care program and reimbursement. You must ensure that all items are completed in order to be entitled to a refund (…). Yes and at the pharmacy most things are free. But all those papers to get that right are not easy (…)*.’ (p10)). They often get support from informal caregivers or from social assistants of their health care insurance for their financial administration, which they perceive very helpful to overcome the administrative challenges.

Respondents mainly talked about their medical doctors when asked for their perception of the quality of received care. Some respondent remarked differences in treatment between the primary care and hospital setting (‘*The doctor that diagnosed me was an older person. He said that you should wait as long as possible before administering insulin. But here at the university hospital they say the opposite, start administering insulin as soon as possible*.’ (p26)). Participants who had experiences with care in other countries evaluated the care in Belgium as very good, because they perceived that practitioners have more time for patients than elsewhere and that they are more personal. Respondents that were explicitly happy with the quality of care often attributed this to the long duration and the quality of the relationship with their care provider. (‘*It’s important, you have to be something like “a friend” of a patient. The patient feels more sure and respects the doctor more. That is my opinion*.’ (p34) Family members seemed more critical than patients in some quotes (‘*We’re going to be honest Mum, the previous GP wasn’t so alert always*.’ (p19)).

Respondents who were largely treated in hospital settings sometimes referred to educators and nurses in the hospital team as a source of information. Participants interviewed in a multidisciplinary primary care practice setting with GPs, nurses, dieticians, social workers and physiotherapists as part of the care team, experienced this setting to help them tackling the chronic disease. Since respondents said doctors (both GPs and specialists) to provide little information about the complementary role of nurses and educators, they perceived them as less important.

Respondents also did not mention dieticians, podiatrists, physiotherapists and other paramedical care professionals that would be available in a first line setting but that are often outside of their primary care premises. Moreover, when asked if they would go to see a dietician or a diabetes educator, respondents doubted the added value, because they perceived to have enough knowledge *(‘Dietician? I have always worked in a poultry and game factory, so I eat a lot of chicken. Occasionally I eat pork. I eat a fair amount of steak, horse steak. I also regularly run low on sugar. So… [I don’t need a dietician]*.’ (p18)). Participants sometimes indicated that there was a lack of ‘finetuning’ or integration of the approach between all the involved stakeholders in the care network. *(‘I did receive a book about diabetes from the university hospital. The lady from “family care” who cooks doesn’t take it into account, so it’s not ideal either.’ (p27)*

### Daily life and self-management

T2DM has a huge impact on daily life. The interviewees indicated being able to perform fewer activities because of their limited energy level and increased fatigue. Furthermore, the participants indicated that the moment they received the T2DM diagnosis, they felt it hit them hard. Some participants experienced negative emotions as a consequence, which influenced their daily lives. (‘*As I said that was like a bomb shooting at us. So, we knew about her kidneys that they were deteriorating, but this was all new to us*.’ (p10-caregiver)) ‘*Well, I’ve been in a deep hole, you should know that. That’s in your head… that’s destroying your body*.’ (p14)

Knowledge about the course of disease and risks were not present among all participants. Some who had experienced the consequences of the disease admitted they never knew what could happen to them beforehand and some now have feelings of regret. (‘*I always thought that it was a far cry from my situation, that’s not going to happen to me*.’ (p11)) ‘*The problem was that I was not responsible. I did not take it seriously. If now, I could change it, I would do it different, do the diet and the exercises*.’ (p34)) In contrast, some respondents perceived their health to be very good, despite the fact all had been selected for having complications. *(‘No, I am healthy as a horse.’* (p18))

Respondents indicated that they find it difficult to cope with T2DM and the consequences on daily life, and related how self-management of T2DM requires a lot of perseverance. Many participants struggled to follow a healthy diet. (‘*I’m tired of the diet, I don’t need it anymore. That’s when I start eating wrong. I’ll get whipped cream and if I go to the shop on my own, it’s also very difficult*.’ (p11) ‘*It is not easy. I feel like I’m in prison. I am not allowed to eat or drink anything*.’ (p20)) T2DM often gets out of control due to crisis moments in life. For example, after the death or illness of a loved one, the follow-up and self-management of T2DM gets derailed. (‘*I think after dad died, three weeks later, you were emotionally and physically broken. Mum couldn’t stand up anymore. There have been several examinations and they said that it was also due to the emotional aspect […]*.’ (p19-family member))

Furthermore, the participants indicated that several activities of daily life are impacted because of T2DM. Financial burden also influences the self-management of T2DM. Facing financial hardships imply that there is little energy left for self-management.(‘*My disease is influenced by other difficult things in my life. Financial problems make me feel more sick and take the energy away you need to take good care of yourself. (…) When a debt collector is waiting at the door (…)*.’ (p14)).

## Discussion

This qualitative study among patients with T2DM complications aimed to improve understanding how they experience the care they receive in the health system of Belgium. A main observation is that we could not identify one respondent having a perfect journey through the health system as it provides services. Our qualitative study illustrates that even in a well-resourced health system like that of Belgium, for subgroups of patients the optimal treatment during various phases of their disease is a challenge because the health system and its professionals often do not seem to optimally match with the clinical and psychosocial needs of the patient.

Many participants were diagnosed late in the course of their disease, when clear signs were already present. The late timing of diagnosis in the study cohort is of concern since a late diagnosis increases the risk of complications. This led to global recommendations for screening above the age of 40 years [18]. In most European countries including Belgium, screening is opportunistic, meaning that it is only done when people consult a health care provider [19], which misses out on people who are not seeking health care. Community-based screening is known to improve case-finding among high-risk groups [20]. The Flemish pilot programme ‘HALT2DIABETES’ which promotes diabetes screening through a community and web-based information and tools is a good start to reach these people and to prevent long term complications [21].

People appreciate the personal and long-lasting contact with a medical doctor because they experience it to be closer and more supportive. The contact with and role of paramedical providers was less recognized especially when these were not part of the core treatment team like in some monodisciplinary primary care facilities, compared to the multidisciplinary ones, which also exist. The patients’ positive perception of relationship with the central health care provider as a person is consistent with other Belgian studies [7]. Patients experience a high level of emotional support and empathy which positively influence the patient’s understanding of their condition. The positive role of relational support to self-management has also been confirmed in other studies, where patients wished to explore feelings and share emotions with professionals [22].

However, not all respondents seemed to appreciate the multidisciplinary and integrated approach in which different professionals and the patient collaborate. This illustrates the need to make patients more aware of the team approach, collaboration and the complementary role of paramedical professionals such as nurses, dieticians and physiotherapists. Paramedical professionals have a role in tailoring information to the context of the patient, and in increasing health literacy. Their added value towards quality of diabetes care is proven in the Belgian context [23]. Additionally, the integration of a social care worker in the health system who can help patients with the administrative and financial burden would be beneficial. A good relationship between patients and their GPs could facilitate this process, because patients often recognize their GPs as the ‘coordinator’ of their care [24]. While diabetes educators are meant to be central in the self-management support for people with T2DM, the respondents in our study did not note that as such. Potential explanations might be that our respondents – people with advanced disease – consider their medical doctors as more important, but this assumption will need further exploration. Ideally, the patient and his/her informal caregivers have an important role, because active involvement in the therapy increases the chances of a higher level of self-management and quality of life [25].

T2DM care has a significant impact on the financial budget of people in Belgium. Reimbursements and additional benefits are available, but some people experience barriers to access those, often due to administrative complexity. Moreover, non-medical costs are not reimbursed, but are needed and of a higher burden compared to the medical costs. Third, self-management is tedious, and hampered by other worries that people may have such as financial constraints and life-time events. We will relate these findings with other studies, and discuss the implications for the Belgium health care system.

The financial impact of out of pocket payments among T2DM patients with financial constraints, is consistent with studies showing the relatively poor performance of the Belgium health systems in terms of equity [5,8] as existing patient care pathways packages are sometimes not known to people and not all costs are covered. In many countries, exclusion from social protection is a challenge [26]. A combination of general population measures (reducing poverty, decreasing user fees) and target schemes with sufficient attention for how they reach the most vulnerable remains a point of attention in the near future [27].

Education to better self-manage one’s disease is widely available but does not reach everybody effectively. Despite much information being available in different formats, people expressed low awareness about the course of diabetes, risks of complications, care and management they needed and the support available. Our findings are consistent with other studies that show that health literacy is hampered also by organizational and structural barriers [28]. This implies that optimal information should be understandable, and that health care organizations must adapt to the information needs of people [29]. Health care providers need to be prepared to understand and meet the patient’s barriers, aspirations and goals. Goal-oriented care provides tools for patients and providers to take these matters into consideration, thereby allowing them to discuss T2DM management based on the patient’s perspective [11,30,31]. Information goes beyond medical issues – for example, about options for financial support and reimbursements. For people with multiple vulnerabilities, such as low health literacy, social and financial constraints and difficult family situations, this would require more intensive psychosocial and other support. For patients, paramedical and psychosocial interventions often significantly influence quality of life. Their role cannot only but especially be valuable at turning moments in a patient’s life, to safeguard linkage to care, contribute to more seamless pathways of integrated care.

Care and support from informal caregivers have significant positive (e.g. support) and negative influences (e.g. lifestyle) on the daily lives of patients [32]. Studies have pointed to the added value of, for example, family system approaches and cognitive-behavioral interventions [33]. Including the family in the education and support activities can thus enhance collaboration of the patient and his/her system with the care system as part of integrated care.

There was little homogeneity within the small group of patients in our study in terms of treatment they report to receive and we found health care providers might be at risk to treating comparable patients differently. The phenomenon ‘clinical inertia’ indicates that patients are not started on intensified treatment despite not achieving their treatment goals. This seems to be more prominent among people living in vulnerable circumstances [34]. This can lead to a significant proportion of patients experiencing years of suboptimal glycemic control before treatment is escalated, resulting in prolonged periods of uncontrolled hyperglycemia and increased risk of diabetes-associated complications and reduced life expectancy [35,36,37].

## Strengths and Limitations

The strengths of our study relate to the design and analysis. Our qualitative design with extreme case sampling provided the opportunity to detect the lived experiences of the target group. The inductive thematic analysis enabled exploration and understanding of the meaning of several social phenomena in a familiar setting [37]. Furthermore, by using a semi-structured interview guide starting with open-ended questions, the participants had the opportunity to take the interviewer into their own narratives. The sampling also ensured we collected the experiences of patients from different geographic locations and health service level providers. The limitations of our study relate to the generalizability and to the extent of patient involvement. Although the study is meant to centralize patients’ experiences, we did not involve patients in the design and methods of the study. Their input could have provided useful advice, reflections and relevance, and would have contributed to increasing patient-centered research. The findings of our study are primarily relevant for the health care context of Belgium. Yet, the analysis revealed a number of themes that are recurring in other settings, as shown in the discussion section. Future ethnographic meta-analyses can built upon this work to develop more generalizable lessons.

## Conclusion

In conclusion, this qualitative study conducted among patients with T2DM complications in Belgium showed that the optimal treatment during various phases of the disease is a challenge because the incentives of the health system and its professionals do not always optimally match with the clinical and psychosocial needs of the patient. We suggest to enhance the role of paramedical professionals and integrate a social care worker, reduce financial constraints, and increase health literacy through more patient-centered, goal-oriented care.

## Appendix 1

**Table:**

Opening questions: – Can you tell me for how long you have got diabetes and how it was discovered? – Which health care worker diagnosed you? – How did the disease and the management evolve since then?	

**General topic**	**Specific topic**

General information	Age Job, income and education Receiving of governmental subsidies Family situation and residency

Diseases	T2DM Comorbidities and other problems

Life with T2DM	Priorities in life Barriers in life

Care timeline	Overview of the providers you visit and how often Current treatment Which kind of services are linked to it? Use of health care services Barriers and facilitators Difficulties in attending services Postpone appointments

Financial aspects	Barriers and facilitators Government support Reimbursement of health care interventions Pharmacy and medication Transport to health care providers Expenses and costs related to T2DM Medical care Hospitalisation Other expenses Percentage expenses related to overall income Most expensive items regarding to treatment of T2DM

Home & living environment	Barriers and facilitators

Self-management of T2DM	Auto daily follow-up of T2DM Awareness of the risks and complications of T2DM Nutrition Lifestyle

Non-medical care	Informal care Household assistance

Assistive devices	Medical devices linked to T2DM Home adaptations Small assistive devices Home equipment

Participation in daily life	Environment Activities of daily life Social life

Accessibility and perceived quality of care	Language Distance to care provider Relationship with provider

T2DM: type 2 diabetes mellitus.
